# Clinical and radiographic outcomes of Aloe vera as a pulpotomy medicament in primary teeth: a systematic review

**DOI:** 10.3389/fdmed.2026.1845683

**Published:** 2026-07-03

**Authors:** Samiksha Uchil, Manju R, Meghna Bhandary, Sadhvi B, Urvashi Shetty

**Affiliations:** 1 Department of Pediatric and Preventive Dentistry, AB Shetty Memorial Institute of Dental Sciences, NITTE (Deemed to be University), Mangalore, Karnataka, India; 2 Department of Oral Pathology, AB Shetty Memorial Institute of Dental Sciences, NITTE (Deemed to be University), Mangalore, Karnataka, India

**Keywords:** aloe vera, formocresol alternative, primary molars, pulpotomy, vital pulp therapy

## Abstract

**Introduction:**

Pulpotomy is a common vital pulp therapy in primary molars aimed at preserving radicular pulp vitality until exfoliation. Concerns regarding the cytotoxicity and systemic effects of formocresol have prompted interest in biocompatible alternatives such as Aloe vera, which possesses anti-inflammatory, antimicrobial, and regenerative properties.

**Aim:**

To evaluate the clinical and radiographic success of Aloe vera as a pulpotomy medicament in primary molars compared to conventional agents.

**Methods:**

A systematic search of PubMed, Scopus and Web of Science was conducted in accordance with PRISMA guidelines. Randomized controlled trials evaluating Aloe vera as a pulpotomy medicament in primary molars were included. Risk of bias was assessed using the Cochrane RoB2 tool, and the certainty of evidence for primary outcomes was evaluated using the GRADE approach.

**Results:**

Clinical success at 3–6 months was high (90%–100%) and comparable between groups. However, radiographic success was highly inconsistent; two major trials reported significantly lower success for Aloe vera (21.1% and 54.8%) compared to formocresol at 6–12 months.

**Conclusion:**

Aloe vera demonstrates promising short-term clinical performance, but it showed less predictable radiographic outcomes and was inferior to standard comparators in some trials. Due to the Very Low certainty of evidence and high risk of bias in major studies, herbal agents cannot currently be recommended as definitive substitutes for formocresol or MTA.

**Systematic Review Registration:**

https://www.crd.york.ac.uk/PROSPERO/view/CRD420251230179, PROSPERO, identifier CRD420251230179.

## 1 Introduction

Vital pulp therapy plays a pivotal role in preserving teeth affected by deep caries or traumatic pulp exposure and is performed in both primary and permanent dentition. Among vital pulp therapy procedures, pulpotomy remains the most widely performed technique in pediatric dentistry, aimed at the removal of inflamed coronal pulp while maintaining the vitality of the radicular pulp to ensure normal exfoliation and function (1). The long-term success of pulpotomy is multifactorial and depends on accurate case selection, adequate coronal seal, operator skill, aseptic technique, and the choice of medicament. An ideal pulpotomy medicament should provide hemostasis, antibacterial action, biocompatibility, and the ability to stimulate healing or regeneration of pulp tissues (1, 2).

Historically, formocresol (FC) has been considered the “gold standard” for primary teeth pulpotomy for several decades. However, concerns regarding its potential cytotoxicity, mutagenicity, and systemic distribution have significantly reduced its acceptance in contemporary clinical practice (1, 8). This shift has accelerated the exploration of more biocompatible and biologically favorable alternatives, such as mineral trioxide aggregate (MTA), Biodentine, and herbal-based medicaments (1, 3). Although MTA and Biodentine demonstrate excellent clinical outcomes, their widespread adoption is limited by cost, technique sensitivity, and handling characteristics (3). Consequently, increasing attention has been directed toward natural, accessible, and cost-effective bioactive agents for pulpotomy. Herbal medicaments including propolis, turmeric (*Curcuma longa*), garlic oil (*Allium sativum*), and *Nigella sativa* have demonstrated antimicrobial, anti-inflammatory, and regenerative potential in vital pulp therapy procedures (6, 9, 11).

Among natural medicaments, Aloe vera, a succulent plant widely used in traditional medicine, has gained attention for its anti-inflammatory, antimicrobial, antioxidant, wound-healing, and regenerative properties (4, 6). Phytochemical analyses have demonstrated that Aloe vera gel contains acemannan, polysaccharides, vitamins, minerals, and essential amino acids that contribute to fibroblast proliferation, collagen synthesis, and modulation of inflammatory responses (4, 5). These biological properties make Aloe vera a promising candidate for vital pulp therapy, particularly in pediatric dentistry, where biocompatibility is of paramount importance (4).

Recent *in vitro* and *in vivo* investigations suggest that Aloe vera may inhibit oral pathogenic bacteria, stimulate tertiary dentin formation, and support pulpal healing (4, 5). Several clinical trials on primary teeth have evaluated its effectiveness as a pulpotomy medicament, reporting encouraging clinical and radiographic outcomes (7, 9–14). However, the available evidence remains inconsistent. While some studies have reported outcomes comparable to conventional pulpotomy medicaments such as formocresol and mineral-based materials, others have demonstrated comparatively lower success rates and increased radiographic failures over longer follow-up periods (9–14). Variations in Aloe vera formulation, concentration, method of preparation, follow-up duration, operator technique, and outcome assessment criteria may have contributed to these conflicting findings (9–14).

Accordingly, this systematic review was undertaken to comprehensively appraise and synthesize the available clinical and radiographic evidence regarding the use of Aloe vera as a pulpotomy medicament in primary teeth. By evaluating the methodological quality of the included studies and identifying gaps in the existing literature, this review aims to support evidence-based clinical decision-making and provide direction for future research in biologically driven pediatric pulp therapy.

## 2 Materials and methods

### 2.1 Protocol and registration

This systematic review was conducted and reported in accordance with the Preferred Reporting Items for Systematic Reviews and Meta-Analyses (PRISMA 2020) guidelines. The review protocol was prospectively registered in the International Prospective Register of Systematic Reviews (PROSPERO; registration number: CRD420251230179).

### 2.2 Reviewer selection

Study selection, screening, data extraction, and risk-of-bias assessment were performed independently by reviewers with academic and research experience in pediatric dentistry, dental materials, evidence synthesis, and oral pathology. All reviewers were affiliated with Department of Pediatric and Preventive Dentistry, A.B. Shetty Memorial Institute of Dental Sciences, NITTE DU, an institution recognized for its longstanding contributions to dental education, clinical practice, and research in India.

The review team comprised postgraduate researchers and faculty members from the Departments of Pediatric and Preventive Dentistry and Oral Pathology with experience in pediatric pulp therapy, dental biomaterials, and research methodology. Prior to study selection and data extraction, calibration exercises and structured discussions regarding eligibility criteria, outcome assessment parameters, and risk-of-bias evaluation were conducted to ensure methodological consistency and minimize inter-reviewer variability. Any disagreements arising during the review process were resolved through discussion and consensus.

### 2.3 Focused question (PICO framework)

The research question was formulated using the PICO framework, where the population comprised children with vital, cariously exposed primary teeth; the intervention was Aloe vera as a pulpotomy medicament; the comparator included conventional pulpotomy medicaments; and the outcomes assessed were clinical and radiographic success.

The focused question was as follows:

“Among children with vital, cariously exposed primary teeth, does pulpotomy with Aloe vera, compared with conventional pulpotomy medicaments, result in improved clinical and radiographic outcomes?”

### 2.4 Information sources and search strategy

A comprehensive literature search was independently conducted by two reviewers (SU and SB) across PubMed/MEDLINE, Scopus and Web of Science to identify relevant randomized controlled trials. The initial search was conducted on October 28, 2025, updated on October 31, 2025, and a final search update was performed on June 2, 2026 to ensure completeness and reproducibility of the search strategy.

The search strategy combined Medical Subject Headings (MeSH) and free-text keywords adapted for each database. The primary search strategy included the terms (“Aloe vera” OR “Aloe barbadensis”) AND (“pulpotomy” OR “vital pulp therapy”) AND (“primary teeth” OR “deciduous teeth” OR “primary dentition”).

In addition to the electronic search, manual screening of the reference lists of included articles and relevant review papers was undertaken. Detailed search strategies for each database are presented in Table 1.

**Table 1:** Electronic search strategy.

Database	Search strategy	Filters applied	Search date	Records retrieved
PubMed/MEDLINE	((“Child"[Mesh] OR “Preschool Child"[Mesh] OR “Infant"[Mesh] OR child*[tiab] OR pediatric*[tiab] OR preschool*[tiab] OR infant*[tiab]) AND (“Tooth, Deciduous"[Mesh] OR “Molar"[Mesh] OR “primary molar*"[tiab] OR “deciduous molar*"[tiab] OR “primary teeth"[tiab] OR “primary teeth"[tiab] OR “deciduous teeth"[tiab] OR “deciduous tooth"[tiab] OR “primary dentition"[tiab] OR “milk teeth"[tiab])) AND (“Pulpotomy"[Mesh] OR pulpotom*[tiab] OR “vital pulp therapy"[tiab] OR “pulp treatment"[tiab] OR “pulp dressing"[tiab]) AND (“Aloe"[Mesh] OR “Herbal Medicine"[Mesh] OR “Phytotherapy"[Mesh] OR “Plant Extracts"[Mesh] OR “Aloe vera"[tiab] OR aloevera[tiab] OR “Aloe barbadensis"[tiab] OR acemannan[tiab] OR “herbal medicament*"[tiab] OR “natural product*"[tiab] OR phytotherapy[tiab])	English language	02 June 2026	11
Scopus	TITLE-ABS-KEY ((“primary teeth” OR “primary teeth” OR “deciduous teeth” OR “deciduous tooth” OR “primary dentition” OR “deciduous dentition” OR “primary molar*” OR “deciduous molar*”) AND (pulpotom* OR “vital pulp therapy” OR “pulp treatment”) AND (aloe* OR “aloe vera” OR “Aloe barbadensis”) AND (child* OR pediatric* OR paediatric* OR preschool* OR “young children”))	English language	02 June 2026	7
Web of Science Core Collection	TS((“child” OR “pediatric” OR “preschool”) AND (“primary teeth” OR “primary teeth” OR “deciduous teeth” OR “deciduous tooth” OR “primary dentition” OR “deciduous dentition” OR “primary molar” OR “primary molars” OR “deciduous molar” OR “deciduous molars”) AND (“Pulpotomy” OR “vital pulp therapy” OR “pulp therapy” OR “dental pulp treatment” OR “pulpotomy agent”) AND (“Aloe vera” OR “Aloe barbadensis” OR “Aloe”) AND (survival OR success OR longevity OR “clinical outcome” OR follow-up))	English language	02 June 2026	100

### 2.5 Eligibility criteria

Studies involving children with primary teeth undergoing pulpotomy procedures were considered eligible for inclusion. Only studies evaluating teeth with vital pulp and no evidence of irreversible pulpitis or necrosis were included. The intervention of interest was Aloe vera used as the primary pulpotomy medicament, with comparator groups consisting of conventional pulpotomy agents such as formocresol, mineral trioxide aggregate, Biodentine, or other medicaments. Eligible studies were required to report clinical and/or radiographic outcomes with a minimum follow-up duration of 6 months.

Only randomized controlled trials published in the English language were included. Studies involving permanent teeth, necrotic pulp, adjunctive use of Aloe vera, observational designs, case reports, case series, *in vitro*investigations, animal studies, conference abstracts, editorials, and studies lacking relevant outcome data were excluded. Studies with follow-up periods shorter than 6 months, non-English publications, and unavailable full texts were also excluded.

### 2.6 Study selection

Study selection was performed independently and in duplicate by two reviewers (SU and SB). Screening was conducted sequentially through title screening, abstract screening, and full-text assessment. Any disagreements were resolved through discussion or consultation with a third reviewer (MR). The study selection process is illustrated in the PRISMA flow diagram.

### 2.7 Data extraction

Data extraction was independently conducted by two reviewers (SU and SB) using a pre-designed and pilot-tested data extraction form. Disagreements were resolved through discussion and consensus or consultation with a third reviewer (MR).

The extracted data included author details, year of publication, study design, sample size, participant characteristics, intervention protocols, Aloe vera formulation and application method, comparator medicaments, follow-up duration, outcome measures, and success criteria.

The primary unit of analysis in the included studies was the tooth rather than the patient, since clinical and radiographic outcomes were assessed individually for each treated primary teeth. These methodological characteristics were considered during interpretation of the findings to minimize potential overestimation of treatment effects (Table 2).

**Table 2:** Characteristics of included studies.

Study (author, year)	Design	Sample size/age	Groups compared	Medicament/concentration/source	Pulpotomy procedure	Contact time	Follow-up	Outcome measures	Statistics
Abirami et al. (9)	Double-blinded RCT, 3-arm	50 children (82 molars), 5–9 yrs	1) Formocresol 2) Allium sativum oil 3) Aloe vera gel	Fresh Aloe vera gel; garlic extract; Buckley's FC 1:5	LA → RD → caries removal → pulp amputation → hemostasis 3–5 min → medicament → ZOE → GIC → SSC	5 min	6 & 12 months	Clinical: pain, swelling, sinus; Radiographic: radiolucency, resorption	Chi-square, McNemar; SPSS v23
Jajoo et al. (10)	Randomized clinical trial	39 molars, 5–6 yrs	1) FC 1:5 2) Aloe vera gel 3) EndoSequence	Commercial Aloe vera gel; FC 1:5	Standard pulpotomy: amputation → hemostasis 5 min → medicament → ZOE → SSC	5 min	3 & 6 months	Modified Zurn & Seale criteria (clinical + radiographic)	SPSS v23, *p* < 0.05
Mustafa et al. (12)	Parallel RCT (1:1)	42 molars, 4–7 yrs	1) 70% Aloe vera gel 2) FC 1:5	70% lab-prepared Aloe vera gel	Amputation → hemostasis 3 min → medicament → noneugenol base → GIC → SSC	3–5 min	3, 6, 9, 12 months	Clinical: pain, sinus, mobility; Radiographic: furcation/periapical radiolucency, resorption	Chi-square; SPSS v19
Sharaf et al. (11)	Triple-blinded RCT, 3-arm	66 molars, 4–7 yrs	1) Nigella sativa extract 2) Aloe vera extract 3) FC	Ethanolic extracts of Aloe vera and Nigella sativa	Standard pulpotomy → amputation → hemostasis → medicament → ZOE → GIC → SSC	Standard application	3, 6, 12 months	Clinical: pain, tenderness, mobility; Radiographic: PDL widening, resorption	Fisher's exact; Kruskal–Wallis; R v4.1.3
Divya et al. (13)	Randomized clinical trial	72 molars, 4–10 yrs	1) FC 2) Aloe vera gel	Fresh Aloe vera gel; FC 1:5	Standard pulpotomy → hemostasis 5 min → medicament → ZOE → SSC	5 min	3 & 6 months	Modified Zurn & Seale criteria (clinical + radiographic)	SPSS v20; *p* < 0.05
Putalikar et al. (15)	RCT	60 molars, 4–8 yrs	1) Aloe vera (Lily of the Desert) 2) FC 1:5	Lily of the Desert Aloe vera gel	Amputation → hemostasis ≤5 min → medicament → ZOE → IRM → SSC	5 min	1, 3, 6 months	Clinical: pain, sinus, mobility; Radiographic: resorption, PDL widening	Kappa, Chi-square; *p* = 0.05

### 2.8 Risk of bias assessment

The risk of bias for each included study was independently assessed by two reviewers using the Cochrane Risk of Bias 2 (RoB 2) tool for randomized parallel-group trials. In accordance with current guidelines, the assessment was performed at the level of individual results rather than the study as a whole. Evaluation was structured across the tool's five standard domains: (1) bias arising from the randomization process, (2) bias due to deviations from intended interventions, (3) bias due to missing outcome data, (4) bias in measurement of the outcome, and (5) bias in selection of the reported result.

For trials where the number of treated teeth exceeded the number of participants (Abirami et al., Divya et al., and Putalikar et al.), specific unit-of-analysis considerations were applied. We evaluated whether the original investigators accounted for the clustering effect (intra-subject correlation) through appropriate statistical methods, such as paired or clustered analyses.

Assessors reached a judgment of ‘Low risk’, ’Some concerns’, or ‘High risk’ for each domain. The overall risk-of-bias judgment for a specific result was determined by the least favourable assessment across these domains. For example, Mustafa et al. (2021) was judged as High Risk overall due to high detection bias in Domain 4, as radiographic assessors were unblinded to the materials. Similarly, Divya et al. (2020) was rated High Risk due to significant attrition bias in Domain 3. Any disagreements between the primary reviewers were resolved through discussion or consultation with a third reviewer.

### 2.9 Certainty of evidence (GRADE)

The certainty of the body of evidence for each critical and important outcome was assessed using the Grading of Recommendations Assessment, Development and Evaluation (GRADE) approach. Evidence from randomized trials began with a High-certainty rating and was subsequently downgraded by one, two, or three levels based on concerns within five specific domains: risk of bias, inconsistency (heterogeneity), indirectness of evidence, imprecision of results, and high probability of publication bias.

A critical factor in this downgrading process was the unit-of-analysis issue, which led to consistent downgrading for both Risk of Bias and Imprecision. The primary teeth was used as the unit of analysis across all synthesis levels, yet five of the six included RCTs (Abirami et al., Mustafa et al., Divya et al., Putalikar et al., and Sharaf et al.) utilized the tooth as the primary unit without adjusting for potential clustering effects.

A detailed audit of the participant-to-tooth ratios reveals that this “5 of 6” grouping includes three confirmed clustered studies and two studies with unclear reporting:

- Confirmed Clustered Studies: Abirami et al. (82:50 ratio), Divya et al. (72:59 ratio), and Putalikar et al. (60:34 ratio) explicitly reported enrolling fewer children than teeth.
- Unclear Reporting: Mustafa et al. and Sharaf et al. were categorized as unclear because they reported tooth counts (42 and 66 molars, respectively) but failed to state the total number of children enrolled.

Because Mustafa et al. and Sharaf et al. did not report participant counts, they were analyzed alongside the confirmed clustered studies as they utilized the tooth as the primary unit of analysis without proving a 1:1 ratio. These five trials relied on standard independent tests, such as the Chi-square or Fisher's exact tests, which assume all samples are independent and do not account for intra-subject correlation in children contributing multiple teeth. Only Jajoo et al. (10) avoided this issue by maintaining a strict 1:1 ratio (39 teeth in 39 children), ensuring true statistical independence.

Consequently, the evidence was downgraded for Risk of Bias due to this analytical error (the incorrect choice of statistical test for correlated data) and for Imprecision because the failure to adjust for clustering results in over-precise *p*-values and artificially narrow confidence intervals that do not reflect the true range of the treatment effect.

The final assessed quality of evidence for most outcomes was categorized as Very Low. These assessments were synthesized into a ’Summary of Findings’ (SoF) table (Table 3), utilizing the final analyzed tooth counts of 338 teeth for short-term and 177 teeth for long-term outcomes. The GRADE assessments directly informed the interpretation of findings, highlighting the distinction between promising short-term clinical success and the inconsistent long-term radiographic stability observed in the updated body of evidence.

**Table 3:** GRADE summary of findings. The certainty of evidence was evaluated using the **GRADE (Grading of Recommendations, Assessment, Development, and Evaluation)** approach. Randomized controlled trials (RCTs) started as **High** certainty but were downgraded based on the criteria below.

Outcome	No. of studies/teeth (analysed)	Direction of effect	Certainty (GRADE)	Rationale for rating
Clinical success (3–6 months)	6 RCTs (All primary studies) — 338 teeth (1)	No significant difference (90- 100% success across most groups).	Very Low ⊕◯◯◯	Downgraded three levels: 1. Risk of Bias: Significant unit-of-analysis errors in 5/6 RCTs and high risk in major trials (Mustafa and Divya). 2. Inconsistency: Notable drop in success in long-term trials (Mustafa). 3. Imprecision: Small sample sizes in individual trial arms.
Radiographic success (6–12 months)	6 RCTs (All primary studies) — 338 teeth (1)	Inconsistent results. Major trials (Mustafa, Divya) showed significantly lower success for *Aloe* (21- 55%) compared to standard controls.	Very Low ⊕◯◯◯	Downgraded three levels: 1. Risk of Bias: Significant detection bias (unblinded radiographic assessment) and attrition bias. 2. Inconsistency: Success for *Aloe* ranged from 21.1% to 93% across trials. 3. Imprecision.
Overall success (12 months)	3 RCTs (Abirami, Mustafa, Sharaf) — 177 teeth (2)	Inconclusive. Formocresol generally maintained higher success at one year (62.5- 84.6%) compared to varied *Aloe vera* formulations (21.1- 84.6%).	Very Low ⊕◯◯◯	Downgraded three levels: 1. Risk of Bias: Concerns in concealment and unblinded assessment. 2. Indirectness: Varied herbal formulations (fresh gel vs. ethanolic extract). 3. Imprecision.
Adverse events (internal resorption)	Reported variably across all studies	No serious systemic harms; internal resorption was a common radiographic failure in both groups.	Very Low ⊕◯◯◯	Downgraded 3 levels for Risk of Bias: Evidence comes from trials with high attrition (Divya) and detection bias (Mustafa), Inconsistency: Significant variability in reported rates, ranging from 0% (Abirami) to 26.3% (Mustafa), and Imprecision: The total number of observed events is very low, making the estimate of risk fragile

**Denominators:** The short-term denominator (338 analyzed teeth) represents the final sample available for assessment of clinical and radiographic success at 3–6 months across all six included studies, after accounting for 23 dropouts from the 361 enrolled primary molars. The 12-month denominator (177 analyzed teeth) is lower because only three studies (Abirami et al., Mustafa et al., and Sharaf et al.) reported outcomes at 12 months, whereas the remaining studies provided follow-up only up to 6 months.

**GRADE Explanatory Notes.**

Note 1. Risk of Bias (General & Analytical Limitation) Downgraded by one level for serious risk of bias. Two studies were judged as High Risk: Mustafa et al. (12) due to unblinded outcome assessment and Divya et al. (13) due to a cumulative attrition rate of 15.3% (11/72 teeth). Additionally, five of the six trials (excluding Jajoo et al. (10)) were identified as having a methodological analytical limitation because they applied standard independent statistical tests to data with confirmed clustering or unclear independence without adjusting for intra-subject correlation.

Note 2. Inconsistency Downgraded by one level for serious inconsistency. Success rates for *Aloe vera* demonstrated extreme variability across the trials, ranging from 21.1% in Mustafa et al. (12) to 93.3% in Putalikar et al. (15).

Note 3. Imprecision (Small Optimal Information Size) Downgraded by one level for serious imprecision. The evidence base failed to meet the optimal information size (OIS) requirements, relying on a small total sample of 338 analyzed teeth across six heterogeneous trials. The limited number of outcome events, particularly for long-term radiographic stability, results in wide confidence intervals and unstable effect estimates.

Note 4. Publication Bias Downgraded by one level for suspected publication bias. The evidence base consists exclusively of small, heterogeneous trials with significant variability in *Aloe vera* preparation methods (ranging from fresh mucilage to ethanolic extracts) and application protocols.

Note 5. Triple Downgrading (Adverse Events) The certainty of evidence for Adverse Events (Internal Resorption) is rated as Very Low due to the cumulative impact of three levels of downgrading: serious risk of bias (unblinded assessments), serious inconsistency (varied reporting across 6 months), and serious imprecision (extremely low event numbers).

### 2.10 Inter-rater reliability

Calibration exercises were conducted prior to study selection, data extraction, and risk-of-bias assessment. Agreement between reviewers was assessed using Cohen's kappa statistic, with a value of ≥0.80 considered acceptable.

### 2.11 Data synthesis

Due to substantial clinical and methodological heterogeneity among the included studies, quantitative meta-analysis was not considered appropriate. The observed heterogeneity primarily resulted from variations in Aloe vera formulations and concentrations, comparator medicaments, pulpotomy protocols, outcome assessment criteria, follow-up duration, and reporting methods. Additionally, the limited number of eligible randomized controlled trials and differences in study design reduced the feasibility of meaningful statistical pooling.

Therefore, a narrative synthesis was undertaken to qualitatively summarize and compare the clinical and radiographic outcomes reported across the included studies. The certainty of evidence was evaluated using the GRADE approach, and the summary of findings is presented in Table 3.

## 3 Results

### 3.1 Study selection

**Figure 1:**
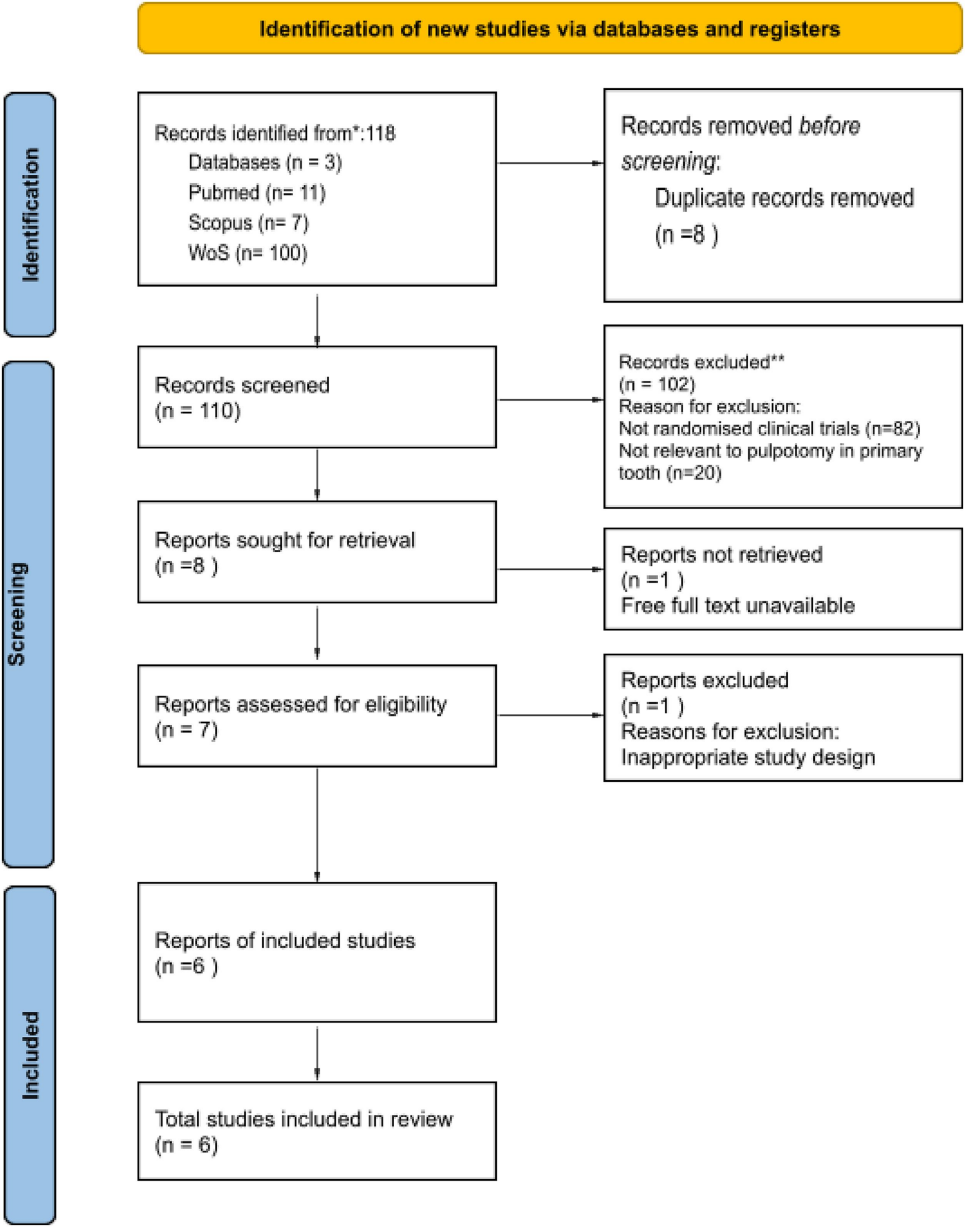
Prisma flowchart. No automation tools were used for screening, and two reviewers (SU, SB) used the RAYYAN screening tool to screen articles for inclusion in the systematic review.

A total of 118 records were identified through the initial database search. Following removal of duplicates and screening of titles and abstracts, full-text articles were assessed for eligibility. Studies excluded at the full-text stage were removed for predefined reasons including non-randomized study design, *in vitro*or animal methodology, absence of Aloe vera as the primary pulpotomy medicament, lack of relevant outcome data, and ineligible population characteristics. Six studies fulfilled the predefined inclusion criteria and were included in the final qualitative synthesis. Detailed reasons for exclusion are presented in the PRISMA flow diagram (Figure 1).

### 3.2 Study characteristics

A total of six RCTs (Abirami et al., Jajoo et al., Mustafa et al., Sharaf et al., Divya et al., and Putalikar et al.) were included, initially involving 361 primary molars at enrollment. The final analysis for short-term outcomes (3–6 months) was performed on 338 teeth, reflecting a total loss to follow-up of 23 teeth across the included trials. The study-specific attrition was as follows: Abirami et al. (82 enrolled, 79 analyzed; 3 lost), Mustafa et al. (42 enrolled, 35 analyzed; 7 lost), Sharaf et al. (66 enrolled, 64 analyzed; 2 lost), and Divya et al. (72 enrolled, 61 analyzed; 11 lost). Jajoo et al. (39 teeth) and Putalikar et al. (60 teeth) reported no dropouts. The denominator for overall success at 12 months was 177 analyzed teeth, as this subset was limited to the three studies (Abirami, Mustafa, and Sharaf) that provided data for the one-year follow-up period.

The included studies evaluated Aloe vera as a pulpotomy medicament against various comparator interventions, including formocresol, Allium sativum, EndoSequence, laser therapy, and mineral-based medicaments such as mineral trioxide aggregate (MTA). Follow-up durations ranged from 6 to 12 months, although some studies additionally reported interim clinical evaluations at 3 months.

Considerable heterogeneity was observed among the included studies with respect to comparator groups, Aloe vera formulation and preparation, clinical protocols, follow-up duration, and outcome assessment criteria. Due to this methodological and clinical heterogeneity, quantitative meta-analysis was not considered appropriate, and findings were synthesized narratively.

### 3.3 Clinical and radiographic outcomes

#### 3.3.1 Clinical success

Across all six included RCTs, clinical success was evaluated in a final analyzed sample of 338 teeth (accounting for 23 dropouts from the initial 361). Most studies reported high clinical success rates for both herbal agents and standard medicaments, ranging from 90% to 100%. No statistically significant difference in clinical performance was observed between *Aloe vera*, Turmeric, and formocresol or MTA at the 6-month interval.

#### 3.3.2 Radiographic success

Radiographic outcomes were assessed in the same 338 analyzed teeth. Unlike clinical success, radiographic results were highly inconsistent. While Abirami et al. and Jajoo et al. reported radiographic success rates exceeding 90% for *Aloe vera*, major trials by Mustafa et al. and Divya et al. showed significantly lower success for *Aloe* groups, at 21.1% and 54.8%, respectively. In these trials, formocresol and MTA demonstrated superior radiographic outcomes compared to herbal preparations.

#### 3.3.3 Overall success (12 months)

Long-term overall success (combined clinical and radiographic) was analyzed in a subset of three RCTs (Abirami et al., Mustafa et al., and Sharaf et al.) totaling 177 analyzed teeth. At the one-year mark, results remained inconclusive. Sharaf et al. reported that *Aloe vera* ethanolic extract maintained a 72.7% radiographic success rate, comparable to formocresol. However, Mustafa et al. observed a significant decline in *Aloe vera* performance over time, concluding that formocresol was superior for long-term radicular pulp preservation.

#### 3.3.4 Adverse events

Internal resorption was the most frequently reported radiographic failure across both herbal and control groups. In studies evaluating Turmeric, the prevalence of internal resorption was notably higher in the powder group (40%) compared to formocresol (6.7%) at 6 months. No serious systemic adverse events were reported in any of the included trials.

Although most studies did not report statistically significant differences between intervention groups, interpretation of these findings should be approached cautiously because of methodological heterogeneity, variations in outcome assessment criteria, and relatively small sample sizes.

### 3.4 Risk of bias assessment

The methodological quality of the included studies was assessed using the Cochrane Risk of Bias 2 (RoB 2) tool across five domains: bias arising from the randomization process (D1), deviations from intended interventions (D2), missing outcome data (D3), measurement of the outcome (D4), and selection of the reported result (D5). Overall, only one study (Sharaf et al.) was judged to have a low risk of bias. Two studies (Mustafa et al. and Divya et al.) were categorised as High risk, while the remaining three studies (Abirami et al., Jajoo et al., and Putalikar et al.) demonstrated Some concerns.

The most frequent methodological concerns related to insufficient reporting of allocation concealment, lack of pre-specified protocols or trial registration, and unblinded outcome assessment for judgment-based results. These methodological considerations were fully integrated into the interpretation of findings and informed the downgrading of certainty in the GRADE assessment.

Detailed Study-Specific Assessments

- Sharaf et al. (11): Judged as Low Risk. This study utilised a computer-generated tool for randomization and numbered opaque sealed envelopes for allocation concealment. It was triple-blinded and provided a pre-registered trial protocol on ClinicalTrials.gov (NCT04719247).
- Mustafa et al. (12): Judged as High Risk. While the study employed rigorous computer-generated randomization, the authors noted that radiographic outcome assessors could not be blinded because the materials were distinguishable on radiographs. According to RoB 2 guidance, unblinded assessment of observer-reported outcomes involving judgment (such as radiograph interpretation) constitutes a High Risk of bias.
- Divya et al. (13) [Subramanyam & Somasundaram]: Judged as High Risk. Significant concerns existed regarding missing outcome data; the text reported a loss to follow-up of 11.1% (eight teeth) at the 3-month evaluation, which introduces critical attrition bias. Regarding attrition in individual trials, **Divya et al.** enrolled 72 molars in 59 children. **At 3 months, 64 molars were evaluated,** while at the 6-month endpoint, 61 molars remained available for analysis. This accounts for the **15.3% cumulative attrition** (11/72 teeth) lost to follow-up over the course of the study. Reporting on the specific mechanism for allocation concealment was also limited.
- Abirami et al. (9): Recorded Some Concerns. Randomization was achieved via a lottery method with sealed envelopes. However, the operator could not be blinded due to differing delivery modes, and reporting on the handling of attrition was limited. No pre-registered protocol was cited.
- Jajoo et al. (10): Evaluated as having Some Concerns. Although computer-based randomization was used, specific mechanisms for allocation concealment were not detailed. Concerns were further raised by the small sample size and the absence of a pre-specified analysis plan or trial registry.
- Putalikar et al. (15): Judged as Some Concerns. The study demonstrated high inter-examiner reliability (kappa = 0.9) and used blinded examiners. However, comprehensive details regarding the allocation concealment mechanism were limited, and no pre-specified trial registry was reported.

**Figure 2:**
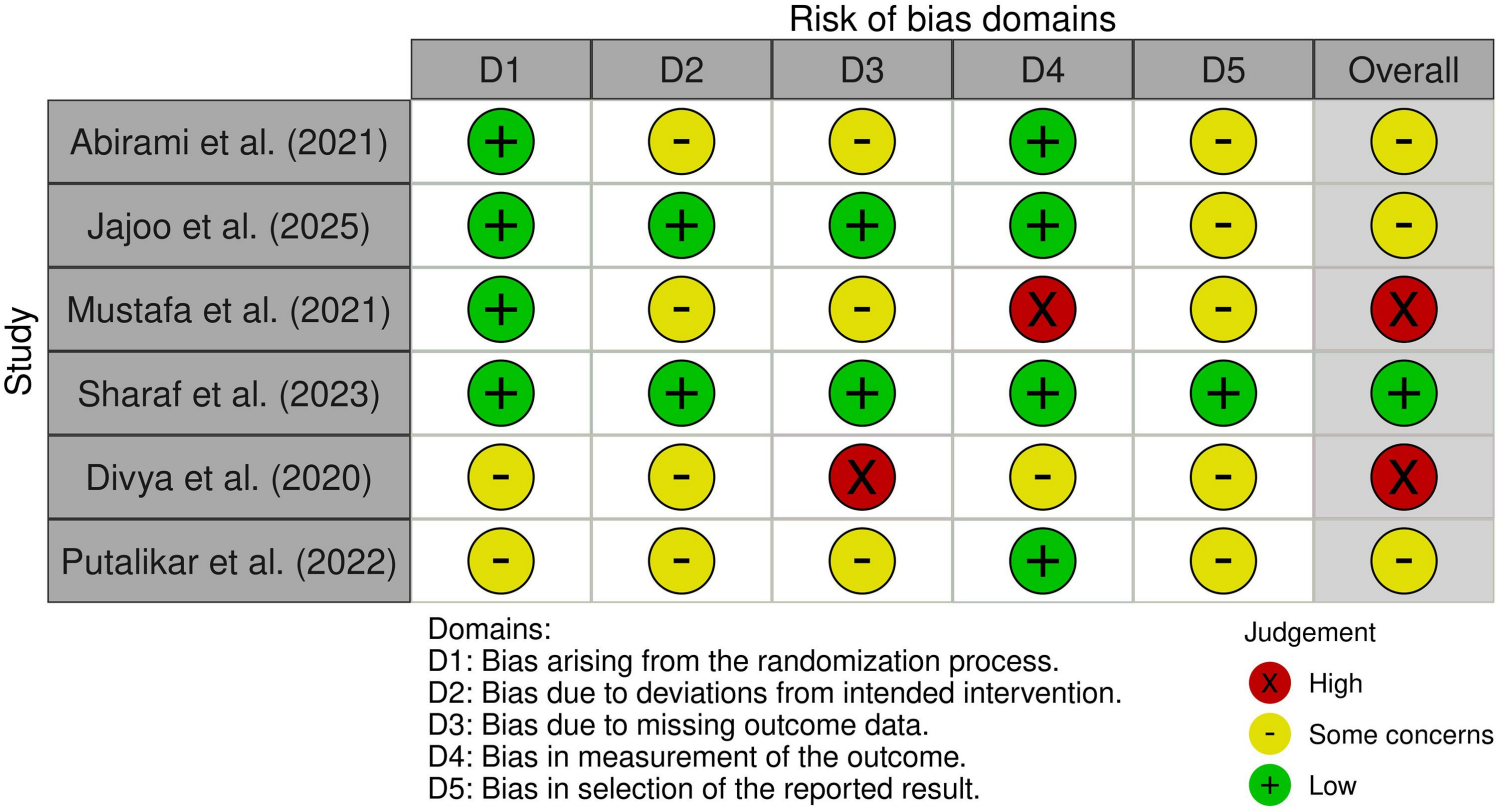
Risk of bias domains.

The certainty of evidence was predominantly Low to Very Low across outcomes, largely due to the identified High risk of bias in major trials, unexplained inconsistency in radiographic success rates, small sample sizes, and the use of the tooth as the primary unit of analysis. A summary of the risk-of-bias assessment is presented in Table 4 and Figure 2.

**Table 4:** Risk of bias assessment.

Study	D1: Randomization process	D2: Deviations from interventions	D3: Missing outcome data	D4: Measurement of the outcome	D5: Selection of the reported result	Overall RoB Judgment	Notes
Abirami et al. (9)	Low	Some concerns	Some concerns	Low	Some concerns	Some concerns	Unit-of-analysis issue: multiple teeth per child analysed using independent tests.
Jajoo et al. (10)	Low	Low	Low	Low	Some concerns	Some concerns	Lacked a pre-specified analysis plan or trial registry.
Mustafa et al. (12)	Low	Some concerns	Some concerns	High	Some concerns	High Risk	High Detection Bias: Radiographic assessors were unblinded to material differences.
Sharaf et al. (11)	Low	Low	Low	Low	Low	Low Risk	Triple-blinded with pre-registered protocol (NCT04,71,9247).
Divya et al. (13)	Some concerns	Some concerns	High	Some concerns	Some concerns	High Risk	High Attrition Bias: Reported a loss to follow-up of 11.1% at 3 months. Unit-of-analysis issue: multiple teeth per child analysed using independent tests.
Putalikar et al. (15)	Some concerns	Some concerns	Some concerns	Low	Some concerns	Some concerns	Unit-of-analysis issue: multiple teeth per child analysed using independent tests.

## 4 Discussion

This systematic review comprehensively synthesized the available clinical evidence regarding the use of Aloe vera as a pulpotomy medicament in primary teeth. The findings suggest that Aloe vera may demonstrate favorable short-term clinical and radiographic outcomes in several studies, with results that were, in certain instances, broadly comparable to those of conventional pulpotomy agents. However, interpretation of these findings requires caution because the currently available evidence remains limited in quantity and characterized by methodological variability and low-to-moderate certainty (7, 9–14).

The biological plausibility underlying the observed outcomes may be attributed to the presence of bioactive phytoconstituents such as acemannan, anthraquinones, and saponins, which possess anti-inflammatory, antimicrobial, antioxidant, and wound-healing properties. These constituents may contribute to modulation of inflammatory responses, promotion of pulp tissue healing, and reduction of postoperative complications (4–6).

The included studies demonstrated variable outcomes regarding the effectiveness of Aloe vera as a pulpotomy medicament. Several randomized clinical trials reported encouraging results. A 2023 randomized controlled trial comparing Aloe vera, *Nigella sativa*, and formocresol in 66 primary molars demonstrated high clinical success rates for Aloe vera, although radiographic outcomes were slightly inferior at 12 months (11). Similarly, a 2020 double-blinded randomized controlled trial involving 72 primary molars reported clinical outcomes comparable to formocresol at both interim and final follow-up evaluations (7). These findings suggest that Aloe vera may provide acceptable short-term clinical performance in primary teeth pulpotomy.

Conversely, a 2017 randomized study comparing Aloe vera with mineral trioxide aggregate (MTA) reported substantially lower success rates for Aloe vera at 12 months, raising concerns regarding the consistency and predictability of its therapeutic performance when compared with well-established bioactive materials (3, 14). Another randomized trial comparing formocresol, Aloe vera, and *Allium sativum* found no statistically significant differences among the treatment groups (9). Collectively, these findings indicate that although Aloe vera may exhibit promising clinical potential, the available evidence remains inconsistent and insufficient to establish it as a predictable alternative to currently established pulpotomy medicaments (7, 9–14).

The variability in findings across studies may be explained by several methodological and clinical factors. Differences existed in Aloe vera formulations, extraction methods, concentrations, storage conditions, application protocols, comparator medicaments, follow-up duration, operator technique, and outcome assessment criteria. Certain studies utilized freshly prepared Aloe vera gel, whereas others employed ethanolic extracts or commercially processed preparations (9–14). Such variations may influence the concentration and bioavailability of active phytoconstituents, thereby affecting the biological activity and antimicrobial efficacy of the medicament (4–6).

Furthermore, differences in restorative protocols and coronal sealing procedures may have independently influenced pulpotomy outcomes regardless of the medicament used (1, 2). Variations in radiographic interpretation criteria and definitions of treatment failure also reduced comparability across studies and likely contributed to inconsistencies in reported success rates (9–14).

Among the reported complications, the most frequently observed radiographic failures included furcal radiolucency, internal root resorption, widened periodontal ligament space, and external inflammatory root resorption. Clinically, occasional cases of pain, swelling, sinus tract formation, and soft tissue abscess were documented. Internal resorption and furcal radiolucency appeared to be the most commonly reported adverse findings. However, meaningful comparison of complication rates across studies remained limited because of differences in follow-up duration and outcome assessment methods (7, 9–14).

The included studies demonstrated certain methodological strengths. Most employed randomized or controlled clinical trial designs and utilized relatively standardized pulpotomy procedures involving hemostasis protocols and definitive coronal restoration. Follow-up durations were generally comparable, ranging from 6 to 12 months, thereby permitting short-term assessment of clinical and radiographic outcomes (7, 9–14).

Nevertheless, several important limitations must be acknowledged. The current evidence base is restricted by the limited number of randomized clinical trials evaluating Aloe vera pulpotomy in primary teeth. Most studies were characterized by relatively small sample sizes and methodological concerns related to blinding, allocation concealment, and incomplete outcome reporting. Considerable variability also existed in Aloe vera formulations, comparator agents, and success criteria, thereby reducing confidence in interpretation of the findings (7, 9–14).

Additionally, follow-up periods were relatively short, limiting assessment of long-term pulpal vitality and tooth survival. Variability in radiographic interpretation methods and the absence of universally standardized outcome criteria further weakened comparability across studies. Restriction to English-language publications may also have introduced selection bias.

Because of the substantial clinical and methodological variability among the included studies, quantitative meta-analysis was not considered methodologically appropriate. Consequently, a narrative synthesis approach was adopted to provide a more clinically meaningful interpretation of the currently available evidence (7, 9–14).

According to the GRADE assessment, certainty of evidence ranged from low to very low across outcomes, reflecting methodological limitations, inconsistency in findings, and imprecision due to small sample sizes. Hence, the conclusions of the present review should be interpreted cautiously.

### 4.1 Unit-of-Analysis considerations

In the included studies, the tooth rather than the patient was generally used as the primary unit of analysis. However, this approach introduces a potential unit-of-analysis issue where multiple teeth from the same child are included, as outcomes may not be fully independent due to shared biological, behavioral, and environmental factors specific to an individual child.

A detailed audit of participant-to-tooth ratios reveals that our ‘5 of 6’ grouping—representing trials that relied on standard independent tests (such as Chi-square or Fisher's exact) without adjusting for intra-subject correlation—includes three confirmed clustered studies and two studies with unclear reporting. Specifically, Abirami et al. (82:50 ratio), Divya et al. (72:59 ratio), and Putalikar et al. (60:34 ratio) explicitly enrolled fewer children than teeth, confirming a clustered design. Conversely, Mustafa et al. and Sharaf et al. were categorized as ‘unclear’ because they reported tooth counts (42 and 66 molars, respectively) but failed to state the total number of children enrolled. Because Mustafa et al. and Sharaf et al. did not report participant counts, they were analyzed alongside the confirmed clustered studies as they utilized the tooth as the primary unit of analysis without proving a 1:1 ratio or applying adjusted statistics (e.g., GEE or multilevel modelling).

Only Jajoo et al. (10) avoided this issue by maintaining a strict 1:1 ratio (39 teeth in 39 children), ensuring true statistical independence. The failure to account for clustering effects in the other five trials likely resulted in an overestimation of statistical precision, including artificially narrow confidence intervals and potentially inflated statistical significance. Consequently, the success rates reported in these trials were interpreted with caution and contributed to the Very Low certainty of evidence in the GRADE assessment.

### 4.2 Clinical implications

Based on the currently available evidence, Aloe vera may represent a potential biologically derived alternative pulpotomy medicament in selected clinical situations, particularly where preference exists for natural or potentially more biocompatible materials. However, its use should be approached with considerable caution because of inconsistencies in reported outcomes, the limited number of high-quality studies, and the lack of robust long-term evidence. At present, the available evidence remains insufficient to support the routine clinical use of Aloe vera as a standard pulpotomy medicament (7, 9–14).

### 4.3 Future research directions

Further high-quality research is required to establish the long-term clinical reliability and therapeutic potential of Aloe vera as a pulpotomy medicament in primary teeth. Future investigations should emphasize well-designed multicenter randomized controlled trials with larger sample sizes, standardized Aloe vera formulations and application protocols, and longer follow-up durations extending beyond 24–36 months. Moreover, incorporation of histological evaluation alongside standardized clinical and radiographic outcome criteria may enhance the reliability, comparability, and overall quality of evidence across future studies.

## 5 Conclusion

Within the limitations of this systematic review, *Aloe vera* demonstrated some favorable short-term clinical outcomes; however, these findings are inconsistent and based on a small, heterogeneous evidence base. The overall certainty of evidence for its use ranges from low to very low due to significant methodological limitations, small sample sizes, and substantial variability in preparation methods—ranging from fresh mucilage to ethanolic extracts—and follow-up durations.

*Aloe vera* showed less predictable radiographic outcomes and inconsistent long-term radiographic stability compared to established standards like mineral trioxide aggregate (MTA) and formocresol; notably, success rates varied widely from 21.1% to 93.3%. Due to this inconsistency, it cannot yet be recommended for routine clinical use as a standard pulpotomy medicament. Furthermore, there is a persistent paucity of histological data to confirm its long-term effects on human pulp vitality and healing, as most evidence currently relies on animal models or short-term clinical observations.

Definitive conclusions regarding its clinical applicability require further well-designed, standardized randomized controlled trials (RCTs) with larger cohorts and longer follow-up periods to substantiate its efficacy and safety against established standards. In conclusion, due to the Very Low certainty of evidence and the less predictable radiographic outcomes observed across the six included trials, *Aloe vera* is not yet recommended as a routine substitute for standard medicaments. The current body of evidence is marked by significant inconsistency, necessitating further trials that utilize adjusted unit-of-analysis statistics to properly account for the intra-subject correlation seen in studies involving multiple teeth per participant.

## Data Availability

The original contributions presented in the study are included in the article/Supplementary Material, further inquiries can be directed to the corresponding author.
